# Assessing selection procedures and roles of Community Health Volunteers and Community Health Management Committees in Ghana’s Community-based Health Planning and Services program

**DOI:** 10.1371/journal.pone.0249332

**Published:** 2021-05-05

**Authors:** Evelyn Sakeah, Raymond Akawire Aborigo, Cornelius Debpuur, Engelbert A. Nonterah, Abraham Rexford Oduro, John Koku Awoonor-Williams

**Affiliations:** 1 Navrongo Health Research Centre, Navrongo, Upper East Region, Ghana; 2 Policy Planning Monitoring and Evaluation Division, Ghana Health Service, Greater Accra Region, Ghana; Waikato Institute of Technology, NEW ZEALAND

## Abstract

**Background:**

Community participation in health care delivery will ensure service availability and accessibility and guarantee community ownership of the program. Community-based strategies such as the involvement of Community Health Volunteers (CHVs) and Community Health Management Committees (CHMCs) are likely to advance primary healthcare in general, but the criteria for selecting CHVs, CHMCs and efforts to sustain these roles are not clear 20 years after implementing the Community-based Health Planning Services program. We examined the process of selecting these cadres of community health workers and their current role within Ghana’s flagship program for primary care–the Community-based Health Planning and Services program.

**Methods:**

This was an exploratory study design using qualitative methods to appraise the health system and stakeholder participation in Community-based Health Planning and Services program implementation in the Upper East region of Ghana. We conducted 51 in-depth interviews and 33 focus group discussions with health professionals and community members.

**Results:**

Community Health Volunteers and Community Health Management Committees are the representatives of the community in the routine implementation of the Community-based Health Planning and Services program. They are selected, appointed, or nominated by their communities. Some inherit the position through apprenticeship and others are recruited through advertisement. The selection is mostly initiated by the health providers and carried out by community members. Community Health Volunteers lead community mobilization efforts, support health providers in health promotion activities, manage minor illnesses, and encourage pregnant women to use maternal health services. Community Health Volunteers also translate health messages delivered by health providers to the people in their local languages. Community Health Management Committees mobilize resources for the development of Community-based Health Planning and Services program compounds. They play a mediatory role between health providers in the health compounds and the community members. Volunteers are sometimes given non-financial incentives but there are suggestions to include financial incentives.

**Conclusion:**

Community Health Volunteers and Community Health Management Committees play a critical role in primary health care. The criteria for selecting Community Health Volunteers and Community Health Management Committees vary but need to be standardized to ensure that only self-motivated individuals are selected. Thus, CHVs and CHMCs should contest for their positions and be endorsed by their community members and assigned roles by health professionals in the CHPS zones. Efforts to sustain them within the health system should include the provision of financial incentives.

## Background

Community participation is a significant part of health service delivery in low-income countries. *Community participation* is a “strategy that provides people with the sense that they can solve their own problems through careful reflection and collective action” [1]. Community health volunteers have been used in other countries to encourage community participation and to augment the work of health professionals [1–3]. The 1978 Alma-Ata Conference reiterated the goal of ‘Health for All by the Year 2000 and approved Primary Health Care (PHC) as crucial to attaining this goal [1].

Evidenced-based intervention programs have proven the importance of community participation in PHC [2–8]. The Bamako Initiative implemented to increase access to PHC by engaging village committees in health delivery management led to improved child health [1]. Haines et al. reported how community health workers contributed to high child survival coverage and other health programs and supported the assessment of the role communities played in increasing coverage of essential interventions [4]. Thus, the success of community-based interventions demonstrated the critical role communities could play in health service delivery in sub-Saharan Africa.

The Community-based Health Planning and Services (CHPS) program was established in 2000 to increase availability and access to basic health services, and Community Health Officers/Community Health Nurses (CHOs/CHNs) collaborate with Community Health Volunteers (CHVs) to provide health services at the doorsteps of rural communities [9, 10]. This cadre of health workers has impacted positively on health service delivery in rural areas in Ghana by assisting in the provision of basic health services including client/patient referral [9]. CHVs are non-salaried community members who are trained to support CHOs in providing basic services in a CHPS zone [11]. The Community Health Management Committees (CHMCs) are constituted to mobilize resources for the building and maintenance of the CHPS compounds, and they support the CHOs and supervise the CHVs activities. CHVs and CHMCs have been trained and integrated into the CHPS program [9]. The CHPS program is jointly implemented by the Ghana Health Service (GHS) and the communities in Ghana. A CHPS zone is a geographical coverage area for community services with a population of 3000 to 4500 people, covering two-to-three-unit committees of the district assembly. The overall strategic goal of CHPS is to improve the health status of the population by strengthening the health system and empowering communities and households for service delivery and utilization [12].

Social research has shown the effectiveness of community health workers in health service delivery in low-income countries. Studies have also revealed how these cadres of health workers have helped improve equitable care and extended access to hard-to-reach populations [10]. The Danfa Comprehensive Rural Health and Family Planning project was successful in using village health workers to deliver PHC services in Ghana [13]. The Bamako Initiative designed to increase access to PHC implemented a minimum integrated health package and based on the concept of community participation, village committees were engaged in health delivery management, which resulted in improved child health [14]. Other studies have justified the continuous use of community health workers in service delivery in sub-Saharan African countries and elsewhere by demonstrating their effect on access and use of health services [2–5, 15–18].

Selecting CHVs and assigning them roles, have been considered a critical process for effective service delivery and sustained volunteer’s activities [19, 20]. Studies have indicated the need for clarity of roles and standardized procedures for selecting CHVs to ensure program impact [21]. The need for selecting CHVs is to encourage broad community participation and holding community elections have been described as the most desirable ways of picking volunteers as health workers [22]. According to Ashraf et al (2013), recruiting CHVs from their communities is important because of local knowledge and a sense of community responsibility to provide health services to the poorer, vulnerable, and underserved populations [18]. Brenner et al. (2013), revealed methods used to select CHVs to include community selection or self-nomination and confirmation by community members [22]. Existing literature shows the diversity of roles and responsibilities of health workers within and across countries [16, 18–21].

Nevertheless, some researchers identified inconsistency in reporting of the selection and roles of CHVs [21]. CHPS is a national strategy that delivers essential community-based health services involving planning and service delivery with the communities. However, the implementation of CHPS is fraught with several policies and systems-level challenges [11]. According to the Ministry of Health, the program is bedeviled with different viewpoints to a lack of clear policy direction and an unending conceptual debate [11]. For instance, at present, community participation within CHPS understood by the role played by CHVs, CHMCs, and other community members are yet to be fully realized. The criteria for selecting CHVs, CHMCs, and the roles these cadre of health workers play and the efforts to sustain these roles are not clear 20 years after implementing CHPS. We, therefore, conducted this research to document current processes in selecting and assigning roles to CHMCs and CHVs and to make suggestions for standardization and sustainability of these roles within the primary health care system in Ghana.

## Study methods

### Methodology

This was an exploratory study design using qualitative methods to appraise the health system and stakeholder participation in Community-based Health Planning and Services program implementation in the Upper East region of Ghana. We conducted 51 in-depth interviews and 33 focus group discussions with health professionals and community members.

### Study setting and design

The study was conducted in seven out of the then thirteen districts (Kassena-Nankana West District, Builsa North District, Bongo District, Talensi District, Bawku Municipal, Pusiga and Garu-Tempane District) in the Upper East Region (UER). The UER is located in the north-eastern corner of Ghana, bounded by Burkina Faso to the north and the Republic of Togo to the east. It covers an area of 8,842 square kilometers. The region’s population according to the 2010 census was 1,046,545 and the population is predominately rural (79%) [23], and there are 259 functional CHPS zones, 286 CHOs/CHNs, 355 CHMCs and 2,173 CHVs in the CHPS program in the region [24].

The Ghanaian public health care system has five levels of providers that include teaching hospitals, regional hospitals, district hospitals, sub-district health centers health posts and health clinics and CHPS zones [25]. The four last levels are represented in the Upper East region. Ghana decentralized its health system in the 1980s, shifting some control to the districts, which are supposed to handle health planning and budgeting [26].

The Ministry of Health is responsible for policy formulation, coordination between public and private sectors, and collaboration with external agencies, and the GHS develop appropriate strategies and set technical guidelines to achieve Ghana’s national policy goals/objectives. The Service also undertakes management and administration of the overall Ghana health resources within the service, and promotes a healthy mode of living and good health habits by people in Ghana [27].

This was an exploratory study design using qualitative methods. We conducted focus group discussions (FGDs) and in-depth interviews (IDIs) with health professionals (District directors of health services, sub-district heads, public health nurses, CHOs/CHNs, and midwives) and community stakeholders (traditional leaders, opinion leaders, women and men leaders, pregnant women, husbands, mothers-in-law, women, and men).

We used a multi-stage sampling method to sample the respondents for the study. First, we divided the districts into East, Central, and West zones. The East zones covered five districts while the Central and West zones covered four districts each. We randomly selected three districts from the East zone (Bawku Municipal, Pusiga and Garu-Tempane districts), two districts (Bongo and Talensi districts) in the Central zone and two districts (Kassena-Nankana West and Builsa North Districts) in the West zone. In each sampled district, 1 CHO/CHN and 1 midwife were randomly selected for the interviews. We then purposively sampled individuals and groups linked to the CHPS program or considered to have information about the program and invited them to participate in FGD or an IDI. These individuals included chiefs, community volunteers, women leaders, pregnant women, nursing mothers, traditional birth attendants (TBAs), district directors of health services, public health nurses, CHMCs, CHVs. We conducted IDIs with the health professionals, CHVs, CHMCs, women leaders, pregnant women, mothers-in-law, traditional leaders, opinion leaders, men, women, and TBAs. Community key informants helped with the identification of men and women above 15 years, nursing mothers, and pregnant for the FGDs (Table 1).

**Table 1:** Sampling approach.

Sampling Approach	Zone		
	East (5 Districts)	Central (4 Districts)	West (4 Districts)
Randomly Selected	3 Districts	2 Districts	2 Districts
Randomly Selected from each district	Midwives	Midwives	Midwives
	CHO	CHOs	CHOs
Purposively sampled from each district	CHVs	CHVs	CHVs
	Chair CHMC	Chair CHMC	Chair CHMC
	Traditional leaders	Traditional leaders	Traditional leaders
	Pregnant women	Pregnant women	Pregnant women
	Mothers of children <5	Mothers of children <5	Mothers of children <5
	Men >15	Men >15	Men >15
	Women >15	Women >15	Women >15
	District Director	District Directors	District Director
	Public Health Nurses	Public Health Nurse	Public Health Nurse
Selected FGD respondents through community key informants	Men and women above 15 years,	Men and women above 15 years,	Men and women above 15 years,
	Nursing mothers	Nursing mothers	Nursing mothers
	Pregnant women	Pregnant women	Pregnant women

#### Training and data collection

We recruited 18 graduate research assistants who had three years’ experience in qualitative data collection and were proficient in at least one of the local languages of the study areas to conduct the interviews and discussions. They were trained for one week on the objectives and methods of the study, the content of the data collection tools, interviewing, and moderating FGDs. We translated key terms in the guides into the local languages (Kasem, Nankam, Buli, Frafra, Mampruli, and Kusali) for consistency of data collection among interviewers. As part of the training, field pre-tests were conducted in districts excluded from the study to test the relevance and appropriateness of the questions and to allow research assistants to practice their interviewing skills.

We collected data from 16^th^ May to 30^th^ June 2017. We included 51 IDIs and 33 FGDs in this study (Table 2). The data collection process required making prior appointments with respondents before conducting the interviews. A four-member team was formed in each district for the field activities. Each district team comprised of a supervisor and 3 interviewers each for the FGDs and IDIs. The district supervisor provided oversight responsibility during data collection. They assisted data collectors in locating sampled communities and organized FGDs and IDIs. As much as possible, the principal investigator and the co-investigators and supervisors observed FGDs, IDIs, and interviews administered and offered suggestions when necessary. The participants in the FGDs ranged from 8–10. All IDIs were conducted either in the homes or workplaces of the respondents, whereas FGDs were conducted in places convenient to the participants and the interviews lasted 45 minutes on average.

**Table 2:** Data collected.

Type of Interview	Category of respondent/Participant	No. of interviews conducted	Total
IDIs	**Health Professionals**-District Directors of Health Services, sub-District heads, Public Health Nurses, CHOs/CHNs, Midwives,	25	51
	**Community health workers**- CHMCs, CHVs and TBAs	8	
	**Community stakeholders**-Community leaders, opinion leaders, mothers-in-law, women leaders, men and women	18	
FGDs	Pregnant women	7	33
	Mothers of children less than one year	8	
	Men and women 15–24, 25–35, 36–50, 51–55	18	
Total			84

Although the number of interviews were predetermined, they were estimated to ensure that saturation would be achieved. Saturation was ensured by constantly comparing data collected to previous data until the data became repetitive [28].

We collected data using open-ended interview guides, designed to elicit themes related to the selection and roles of CHVs and CHMCs. Theoretical sampling allowed for the inclusion of new respondents such as members of DHMTs and the constant comparative approached enabled the research team determine the points of thematic saturation. Daily debriefings with field staff across the research sites enabled the research team refine the data collection tools as data collection progressed and to determine at what point new data was not forth coming.

#### Qualitative data analysis

Interviews and discussions involving community stakeholders were in the local languages while those with the health providers were in English. We, audio-recorded all interviews and discussions and transcribed them verbatim into English. The transcripts were reviewed for accuracy and completeness and corrected to facilitate the work of the coding by theme. The Principal Investigator (ES) and two other Co-Investigators (RA and CD) sorted the transcripts by sources and conducted multiple readings, writing memos in the margins of the text in the form of short phrases, ideas, or concepts arising from the texts. We used these memos to iteratively develop coding categories. Using thematic analysis, we closely examine the data to identify common themes–topics, ideas and patterns of meaning that come up repeatedly and themes that were atypical in response to each question. Transcripts were imported into NVIVO 11.0 for open, axial, and selective coding by three separate coders (ES, RA, and CD). Coders met regularly to discuss the process of coding, revise the codebook as necessary, and resolve any uncertainty in coding. The themes were used to generate reports that allowed us to describe the thoughts and opinions within the interviewee group (e.g., community stakeholders) as well as compare responses across groups (e.g., community stakeholders and health professionals).

#### Ethics approval and consent to participate

We obtained ethics approval from the Institutional Review Board of the Navrongo Health Research Centre (NHRCIRB262). We also obtained written informed consent from all interviewees prior to initiation of the interviews.

Community approval was obtained from chiefs, elders, and compound heads of the study districts before the study commenced. We also assured participants of anonymity and confidentiality before conducting the interviews.

## Results

A total of 33 FGDs with pregnant women, nursing mothers and men and women and 51 IDIs were conducted. Sixty three percent of the IDIs respondents were females (Table 3), and the majority of the FGD participants were Christians (Table 4).

**Table 3:** Demographic characteristics of IDI participants, Upper East region 2017.

Interviews in seven Districts	Number of Indepth interviews	Gender	Gender profile or role in the community
	4	Female	Mother-in-law
	2	Female	Community Health Volunteer
	4	Female	District Director of Health Services
	3	Male	District Director of Health Services
	4	Male	Opinion Leader
	2	Female	sub-District Head
	3	Male	Sub-District Head
	7	Female	Public Health Nurse
	4	Female	Traditional Birth Attendant
	4	Male	Husband of a woman with a child less than one year
	3	Female	Women’s Group Leader
	3	Female	Midwife
	2	Male	Community Health Management Committee Member
	3	Female	Community Health Officer
	3	Male	Community Leader

**Table 4:** Demographic characteristics of FGD participants, Upper East region 2017.

Seven Districts	FGD Number	Total Participants	Gender	Age-range, years	Gender Profile
	2		Females	18–24	
	2	4	Males		
	2		Females	25–35	
	2	4	Males		
	3		Females	36–50	
	3	6	Males		
	3		Females	51–55	
	1	4	Males		
	7	7	Females		Pregnant women
	8	8	Females		Nursing mothers

### Selection of community health volunteers

The selection of volunteers is often initiated by providers in the community health compounds and implemented by the community leaders. The providers usually give a selection criterion which is often adhered to by the community leaders. A member of the DHMT in the Bawku Municipality outlined the procedure for the selection as follows:

*…*.*” That is what I told you that the first thing is to do community entry and discuss with them about the CHVs*, *why you need them*, *and the caliber of persons to be selected as CHVs*. *Normally you make sure that the CHVs are residing in the community and you have to be gender-sensitive; making sure that you have both sexes included and are prepared to stay in the community for at least a reasonable number of years*. *After the community has selected them for you*, *you introduce them back to the community to confirm that they are those that they have selected to work in the community as CHVs before you train them and provide them with the necessary logistics to start work*.*”* **(IDI, DHMT staff, Bawku Municipal)**

Selected volunteers are usually out-doored during a community durbar for community endorsement. “Durbars’ are traditional gatherings comprised of drumming, dancing, speechmaking and public debate” [9]. Community objections of volunteers are noted during this function and where necessary replacements made.

In other communities, the influence of the provider at the community health compound is limited to merely outlining the responsibilities that the volunteer is expected to play, but the criteria for selecting the volunteers are reserved for the community as stated by a member of the DHMT in the Builsa North district.

*…*.*” Yes*, *the criterion was thrown to the community*. *We are here as health professionals to serve you but we cannot do it all alone*. *We need community participation*. *So how do we get them*? *The community considers those they think can do the work for them*. *And through their ‘Kanbong Naabas’ (Sectional chiefs) and sub-chiefs*, *and assemblymen*, *they all come together and identify those people they think they can work well*. *So*, *we cannot impose it on anybody in the community*. *The community with the assemblyman*, *the sub-chiefs*, *and the ‘magazias’ (women leaders) come together and identify an individual they think can help the community and is ready to work until the person proves otherwise*. *That is how we came by the thirty-six (volunteers)*.*”* **(IDI, DHMT staff, Builsa North District)**

In some communities, the position of community volunteers is advertised.

*…*.*” They did not go round to select people*. *What they did was that they advertised that they needed people who could read and write and would volunteer to do free services to their own people and the interested persons went and registered*. *The criterion is that all interested persons must be hard working*. *So*, *if you are hardworking and willing to render free services to your own people*, *you are qualified*. (**Opinion leader, Bawku Municipal)**

Although advertisements were not common in the various districts, it was a useful approach as it ensured that only self-motivated individuals, who were willing to work for free, offered themselves for the job.

Some volunteers also inherited the position from their parents mainly through apprenticeship after the father or mother passes away or becomes too old to perform the duties. This quote from a member of the DHMT attests to that.

*…” Just as I mentioned*, *the elders selected them and some of them*, *their fathers were volunteers so when they become old that they cannot do the work again; he tells you that now I cannot work*, *I want to rest and I want my child to take over*.*”* **(IDI, DHMT staff, Pusiga District)**

Regardless of who suggests the selection criteria, volunteers are usually people who are respected and trusted within their communities. Some communities, however, have additional criteria as the quote below suggests.

*…*.*” The way it was*, *they considered those whose education was higher to be able to do the job and community members with good character*, *people who were enthusiastic and hardworking*, *stable and available to do the work and have community spirit*. *These were the critical criteria they used to select the volunteers*. **(FGD, Men, 18–24 years old, Garu-Tempane District)**

One common criterion that came out across the districts was the ability to speak and write English. Usually, two volunteers are selected to assist health providers. Thus, in some settings, the communities ensure that at least, one volunteer speaks and writes English. This criterion was important because it facilitates interaction between the health providers and the volunteers. In many cases, CHOs do not speak the languages of the communities that they serve and so CHVs often act as interpreters in delivering health information. The role of the volunteers also comes with some documentation which requires writing skills to execute. According to the community members, the volunteers’ ability to read is also important so that they can read instructions on medicines and give the right doses.

In some districts, however, being literate was not necessary for the selection of volunteers, but the emphasis is on being available and willing to work as contained in the excerpt below:

*……” Oh*, *the criteria*, *basically it is somebody who is available at the community level*, *someone who will willingly give their time for the work*. *So*, *there are just two basic criteria we applied*. *We are not worried whether you are literate or an illiterate*, *but we just want your time; whether you are readily available in the community to willingly come and support us when you are needed*.*”* **(IDI, DHMT staff, Builsa North District)**

### Formation of the community health management committee

The formation of the CHMC was part of the CHPS initiative. Health providers usually request community leaders to provide them with a group of people who will support them to provide the needed health services to the community and the outcome is the CHMC as contained in the excerpt below.

*……” It was in a meeting I organized for the CHPS zone so we came for that meeting and the CHO said we needed a community health committee to be formed to provide some support to the CHPS and through that*, *we were selected*.*”* (**IDI, CHMC member, Builsa North District)**

*…*.*” We the community members held a meeting and selected them so that they can collaborate with the nurse and discuss community health issues*. **(IDI, Husband, Kuka, Bawku Municipal)**

The members are usually selected during community meetings or durbars organized by the chief. Apart from considering individuals who are honest and respectful, the community members also consider individuals who have good leadership qualities, patient, tolerant, selfless, available, and willing to work for the community without receiving any payment.

*……” They were selected at a community meeting/durbar organized by the chief*. *They looked for a person who will be committed*, *selfless*, *and patient because if you are not careful*, *you cannot do the work*. *We then nominated people among us to be part of the committee*.*”* **(FGD, Women, 51+ years old, Talensi District)**

The health authorities mostly participate in these durbars and meetings with community leaders to outline the role of the CHMC to guide the selection process. Beyond that, they play an observer role as articulated by a member of the District Health Management Team (DHMT) in Pusiga.

*…*.*” When they are going to recruit*, *they invite us there in the form of a durbar; we sit with them and educate them on the sort of people we want for the community health compound and all of them will be there but one person from the DHMT would be there to observe the meeting and come back with the results so that when they have a meeting it will be reported*.*”* **(IDI, DHMT, Pusiga District)**

*……” First of all*, *you have to visit the community and meet with the opinion leaders*, *tell them the purpose of your visit and what you want them to do; for example*, *select CHMC*. *Then you give them the criteria you want them to use and select them for you*. *With time*, *they will select them for you and after the selection*, *you have to introduce them back to the community who will confirm them as CHMC to serve in that CHPS zone*. *After that*, *you train them and supply them with the necessary logistics and ask them to start to work”* **(IDI, DHMT staff, Builsa North District)**

For the community members, this approach to selecting the CHMC members appears to be the only approach since the nurses are usually new and do not have enough information about community members to inform their choice of an individual to be a member.

The CHMCs are typically made up of about 11 to 15 people comprising women group leaders, sectional representatives, and opinion leaders. Landlords, sub-chiefs, youth group leaders also find their way into the committees. Gender representation on the committees is considered in some communities and there is often a conscious effort to have different sections of the village represented. Literacy is not a selection criterion for members except for the recording and financial secretaries.

Selected members of the CHMC are usually introduced to the community by the providers at a durbar. After the community endorses the nominees, the health workers train them on their roles.

*…*.*” With time*, *they will select them for you and after the selection*, *you have to introduce them back to the community who will confirm them as CHMC to serve in that CHPS zone*. *After that*, *you train them and supply them with the necessary logistics and ask them to start to work”* **(IDI, DHMT staff, Builsa North District)**

Individuals are not obliged to accept nominations to serve on the CHMC. Similarly, community members are not obliged to accept the nominees. Therefore, to forestall such disagreements, consultations are usually held behind the scenes to ensure that the individual is willing to take up the responsibility on behalf of the community and that the community members also endorse his/her membership.

### Roles of community health volunteers

Community volunteers are the representatives of the community in the routine implementation of CHPS. They assist in community mobilization and help health providers to administer vaccines as well as the management of minor illnesses. They also help to translate health messages being delivered by the health providers to the people in their local languages.

A number of volunteers that operate within a community varies based on the spread of the community and the workload involved but typically, each community has at least two community volunteers. From the discussions and interviews, not all the volunteers are active and therefore it was always necessary to select more than one so that at every point in time, a volunteer would be available to assist the health provider.

*…*..*”CHVs yes*, *we have them in all our communities*. *We have at least 2 CHVs in the communities that are very large*. *Those communities are divided and each section has a volunteer*. *In all*, *we have about 209 communities in the district*. *Yes*, *it’s obvious that we have some of them that are not very active*, *but in all*, *the majority of them are doing the work we want to be done*.*”* **(IDI, DHMT staff, Garu-Tempane District)**

The community volunteers play several roles within their catchment areas. One of these critical roles is the mobilization of pregnant women for antenatal care (ANC) and mothers of newborns for child welfare clinics. They help pregnant women and their families prepare for deliveries and encourage them to seek postnatal care for themselves and their babies. They also assist those in need of emergency care to get to the next level of care. These roles were mentioned across the study districts.

A community health volunteer summarized their role as follows:

*……” I play a lot of roles in the CHPS program*. *I mobilize the community members anytime there is a meeting*. *I also participate in health promotion and education*. *I even used my own money to buy food for workers when we were building an additional room*. *I also support the community health officer*, *even though not financially*, *I advise him a lot and encourage him to continue with his good work*. *Anytime he is in need of help and he calls upon me*, *I always help him*. *We go to the houses and when we come across a sick child*, *we tell the parents to send him/her to the clinic*. *We assist the CHO in conducting home visits and also give health education to the community*. *Anytime there is a meeting*, *it is we the CHVs who will mobilize the community*.*”* (**IDI, CHV, Bawku Municipal)**

CHVs also assist in the administration of childhood immunizations especially polio and distribution of insecticide-treated bednets. They support mass drug administrations, such as the distribution of ivermectin and albendazole for the control of onchocerciasis and lymphatic filariasis respectively.

*…*..*”I have realized that they distribute mosquito nets to us and administer drugs for elephantiasis in our homes*. *Also*, *they give out some drugs*, *which I cannot remember the name in our homes*, *even when you are not physically present at home; they would leave the drugs behind with your parents to be given to you when you return home*. *They help with the administration of the Cerebrospinal meningitis vaccine*.*”* (**FGD, Men, 25–35 years old, Kassena-Nankana West District)**

Also, CHVs conduct home visits to carry out health education. Key messages usually include the destruction of breeding places for mosquitoes to prevent malaria, encouraging hygienic practices to prevent diarrhea, and ensuring good nutrition especially exclusive breastfeeding for babies. They also provide home treatment for mild malaria as well as first aid for other infectious diseases before referring patients to the next level of care. Community members who fall sick and are reluctant to use health facilities are usually encouraged by the volunteers to do so.

The CHVs also use their local knowledge of the community and its culture and customs to support health providers to deliver health services to the people in a culturally acceptable manner. They are the link between the health providers at the community health compound and the health consumers within the community. They help in disseminating information to the community members and share information on challenges facing the Community Health Compounds (CHCs). They also bring feedback from the communities on services provided at the CHCs and solutions to some of the challenges.

*…*..*”The volunteers also visit pregnant women or women with children up to 5years to check whether they are following the education the health personnel gave to them*. *And also ensure that pregnant women with problems are reported to the appropriate health personnel for a solution*. *I quite remember one woman who refused to go for ANC because she went and they gave her drugs to take*, *and after taking the drugs she fell sick*. *So she said she would not attend ANC again*, *but through their visit (visit of the volunteers)*, *that problem was solved and the woman was able to continue her ANC*.*”* (**IDI-CHMC Member, Builsa North District)**

Health providers directly supervise CHVs within their catchment areas. Non-performing volunteers are usually reported to the community leaders to either encourage or replace them.

### Incentives for volunteers

Respondents reported that some volunteers complained about the lack of remuneration for their work. Consequently, some often threaten to resign their positions but the community leaders usually intervene to prevail on them to rescind their decisions. Some respondents suggested that both the health system and the community should find a way of motivating the volunteers. While some community members said they were not aware of any incentives being given to the volunteers, some said they have been given bicycles in the past to facilitate their movements between compounds. Nobody mentioned any regular cash payments to volunteers but almost all the participants agreed that they deserve some level of cash compensation. A community member lamented about their situation and justified the need to provide CHVs some incentives in the quote below.

*…*.*”They are called to Tongo and Bolga for training*. *They sometimes beg for transport and feeding money*. *They get help sometimes but mostly no*. *But if there could be a system to support them and their families that will be good*. *They travel to impact our lives*, *so they need help*. *That’s how it is*. *If you give something to a child and send him*, *the feeling and commitment to the call is different from when he/she goes empty-handed*. *They need to be encouraged with something to make them more effective*.*”* (**FGD, Women 51+ years old, Talensi District)**

*……” In my opinion*, *the CHVs are doing a lot because I started as a community health nurse and I know the part they play*. *So*, *left to me alone*, *there should have been incentives or they are given some small amount at the end of the month which will motivate them small if not much; that will make them happy and ensure that they stay and work*.*”* (**IDI, DHMT staff, Pusiga District)**

When asked the type of incentive preferred, the community members ranked non-financial incentives first because they felt that within their context, it was more sustainable than financial incentives. Among the non-financial incentives, there was general consensus on bicycles at the top of the priority list followed by public acknowledgment. Beyond that, no other consensus was built around any other incentive.

….” First of all, in order of importance, if the community can come together and get them means of transport like a bicycle to aid their movement in the work they do, secondly, mobilized communal labor to help them in their farms (because they leave their personal work to volunteer for the community) or whatever they do to survive and if that work is done, they will have time to do volunteerism. Lastly, the various health committees can sit down in the Builsa district to plan ways of motivating the volunteers by way of establishing a fund purposely for that. Since they are being appreciated, they will do their work well. So, ranking them, I will start from the bicycle, personal assistance on their farms, and some token [money] for them.” **(IDI, CHMC Member, Builsa North District)**

### Community health management committees

The group is responsible for mobilizing resources for the development of CHPS compounds including the provision of leadership in constructing temporal structures to accommodate midwives so they can stay in the community and provide delivery services.

*…” These people help to monitor the work of the CHO and mobilize resources for the development of the CHPS compound*. *Some of them come together and they are able to put up temporary structures for the midwives to conduct delivery services in CHPS compounds that lack delivery rooms*. *They also ensure that both the CHPS compound and the CHO have security*.*”* **(IDI, DHMT Member, Bawku Municipal)**

The committee also plays a mediatory role where grievances surrounding health care delivery in the communities are channeled through them to providers in the health compound. They work closely with the community health officers to address challenges that the community health compound faces and also to improve communication between the officers and their clients.

*…*.*” We also*, *sit with the nurses and collaborate*. *Sometimes the nurse may say something to a woman*, *which she is not happy with*, *she will complain to the CHMC and the committee will sit with the nurses and advise them on how issues should be handled so that we can progress*.*”* **(IDI, Husband, Kuka, Bawku Municipal)**

Respondents and discussants said the community has the responsibility of responding to calls by the CHMCs regarding meetings, contributions, and other initiatives. The community is also responsible for guiding the conduct of the CHMC members and to ensure that the committee is performing its duty. Community concerns about the community health compound and the services provided are channeled through the committee to the health authorities.

## Discussion

This study assessed the selection procedure and the roles of CHVs and CHMCs in the implementation of CHPS. The selection of CHVs and CHMCs is currently not standardized within the health system but generally individuals who are willing, honest, and hardworking serve in these positions. CHVs lead community mobilization efforts and assist in providing basic health services in rural communities while the CHMCs mobilize resources for the development of CHPS compounds including serving as mediators between communities and the CHOs. Both CHVs and CHMCs receive non-financial incentives for their efforts.

Communities play a vital role in the participation of volunteers in the CHPS program. They identify and select potential volunteers with specific criteria given by health professionals, who usually train and supervise their work. Although many communities use the health provider’s criteria for the selection process, few do advertise the position and the reason is that the selection of volunteers have received the least attention in the CHPS deployment framework. Thus, some communities do not strictly follow the lay down rules in selecting volunteers [11]. Health professionals and community stakeholders ensure the people with the best attributes [10, 29] are selected to assist in providing health services at the doorsteps of their communities. These findings corroborate earlier studies that revealed that communities select volunteers with good attributes such as good character, the spirit of volunteerism, diligence, trustworthiness, and honesty [10, 22, 30–32] to serve them.

Community health management committees are volunteers with different competencies and responsibilities, who guide and mobilize community resources for the planning and delivery of health services [9, 12]. One of the primary roles of CHMCs is the mobilization of resources and the provision of leadership to construct temporal structures for nurses in CHPS zones to provide health services within their catchment areas. They play a mediatory role where grievances surrounding health care delivery in the communities are channeled through them to providers in the health compound and vice versa. These findings corroborate results from a study that revealed that village committees were part of health delivery management and their impact on child health were enormous [14]. Other studies also revealed the significant role health committees play in health facility operations [33, 34].

Volunteerism typically excludes remunerations or payment of compensations. Thus, both CHVs and CHMCs who are considered volunteers within CHPS are not paid or compensated. Some studies have reported that remunerated volunteers are more likely to be effective in performing their duties than unmotivated volunteers [35]. Although participants in our study settled for non-financial incentives based on sustainability, they also advocated for financial incentives. Several studies have reported providing both financial and non-financial incentives for volunteers [10, 20, 29] and others have stated the outcome of these incentives on CHV’s effective participation [10, 36–40] and increased retention [41]. However, financial incentives could create challenges for communities and health systems in Low Middle-Income Countries. According to Bhattacharyya et al (2001), communities and health systems may not be able to mobilize enough resources to pay adequate compensations, consistent or sustain the payments [41, 42]. Key stakeholders noted community establishing a financial incentive scheme for volunteers would therefore require a critical assessment of available sources of funding and sustainability of the funding mechanisms.

### Limitations

This study has a number of limitations that include recall bias. This could have limited the validity of the data because some participants could have forgotten about past events involving community health workers’ selection and participation in the CHPS program. To minimize recall bias, participants were asked to respond to questions they know about the CHVs and CHMCs. Using different local languages to collect the data could also have distorted the presentation of the questions to the respondents. However, the standard training for field workers and supervisors and the translation and back translation of the questions minimized the language bias and the open-ended interview techniques allowed us to capture the views of the respondents in their own words. This study focused on selection procedures and roles of CHVs and CHMCs within the context of the CHPS program and might not be generalizable to other contexts because of the uniqueness of the design and implementation of the CHPS program in the Upper East region. However, our findings are similar to other programs in developing countries that explained the selection and use of community health volunteers in health service delivery. Social desirability and politeness biases may have been a possibility in this work, but study procedures and training protocols were designed to reduce these kinds of biases and interviewers were university graduates who had no links to the delivery of health services. Respondent bias may have occurred since some respondents were directly involved in selecting and assigning health volunteers and giving them roles in the CHPS program. Despite these challenges, it is important to note that the people we interviewed were forthcoming with information about the program. Four evaluation advisors reviewed and examined the research process and data analysis to ensure that the findings are consistent and could be repeated.

## Conclusion/Recommendations

The CHPS program is established by the Government of Ghana to provide basic health services in rural areas. CHVs and the CHMCs are the representatives of the community in the routine implementation of CHPS. They play an important role in the implementation of primary health care by mobilizing communities for health programs and provision of basic healthcare services in rural communities. The selection criteria for this cadre of health workers differ from community to community, but there is the need to make the selection criteria uniform to ensure that only self-motivated individuals are selected. Therefore, CHVs and CHMCs should contest for their positions and be endorsed by their community members and assigned roles by health professionals in the CHPS zones. Regardless of the setting, the selection usually ensures that only people with the volunteerism spirit, patience, tolerance, and selflessness are given the chance to serve. Besides non-financial incentives, motivating CHVs financially could help to improve their participation and retention.

## List of abbreviations used

CHPS: Community-based Health Planning and Services
CHOs: Community Health Officers
CHNs: Community Health Nurses
CHVs: Community Health Volunteers
CHMCs: Community Health Management Committees
TBAs: Traditional Birth Attendants
IDIs: In-depth Interviews
FGDs: Focus Group Discussions
UER: Upper East Region
PHC: Primary Health Care
DHMTs: District Health Management Teams
ANC: Antenatal Care
